# Economic Benefits of Investing in Women’s Health: A Systematic Review

**DOI:** 10.1371/journal.pone.0150120

**Published:** 2016-03-30

**Authors:** Kristine Husøy Onarheim, Johanne Helene Iversen, David E. Bloom

**Affiliations:** 1 Department of Global Health and Population, Harvard T.H. Chan School of Public Health, Harvard University, Boston, Massachusetts, United States of America; 2 Department of Global Public Health and Primary Care, University of Bergen, Bergen, Norway; University of Rennes-1, FRANCE

## Abstract

**Background:**

Globally, the status of women’s health falls short of its potential. In addition to the deleterious ethical and human rights implications of this deficit, the negative economic impact may also be consequential, but these mechanisms are poorly understood. Building on the literature that highlights health as a driver of economic growth and poverty alleviation, we aim to systematically investigate the broader economic benefits of investing in women’s health.

**Methods:**

Using the Preferred Reporting Items for Systematic Reviews and Meta-Analysis (PRISMA) guidelines, we systematically reviewed health, gender, and economic literature to identify studies that investigate the impact of women’s health on micro- and macroeconomic outcomes. We developed an extensive search algorithm and conducted searches using 10 unique databases spanning the timeframe 01/01/1970 to 01/04/2013. Articles were included if they reported on economic impacts stemming from changes in women’s health (table of outcome measures included in full review, Table 1). In total, the two lead investigators independently screened 20,832 abstracts and extracted 438 records for full text review. The final review reflects the inclusion of 124 articles.

**Results:**

The existing literature indicates that healthier women and their children contribute to more productive and better-educated societies. This study documents an extensive literature confirming that women’s health is tied to long-term productivity: the development and economic performance of nations depends, in part, upon how each country protects and promotes the health of women. Providing opportunities for deliberate family planning; healthy mothers before, during, and after childbirth, and the health and productivity of subsequent generations can catalyze a cycle of positive societal development.

**Conclusions:**

This review highlights the untapped potential of initiatives that aim to address women’s health. Societies that prioritize women’s health will likely have better population health overall, and will remain more productive for generations to come.

## Introduction

The World Bank’s 1993 World Development Report (“Investing in Health”) emphasizes that health matters not just intrinsically, but also instrumentally for a society’s development [1, 2]. In 1994, the International Conference on Population and Development (ICPD) convened more than 20,000 delegates from 179 countries to discuss global development challenges. The galvanizing report of the consensus reached at the conference, the “Programme of Action,” was a landmark in articulating and advancing women’s rights [3]. The delegates adopted resolutions about reproductive health rights, gender equality (especially in regard to access to education), and the reduction of maternal and child mortality. The ICPD report acknowledges that these measures are important not simply for social justice, but because they are essential to viable and sustainable global development. That document informed the guiding set of principles for the United Nations Population Fund (UNFPA) and was a precursor to the Millennium Development Goals (MDGs), a doctrine that built on and expanded the “Programme of Action” mandate. More than 20 years have passed since the ICPD, and further research has explored how investments in health and sound health policies foment economic growth and development [2, 4–8] through various channels, including labor and productivity, savings, education, and age structure [1, 7, 9]. What the research has yet to deliver is an explicit investment strategy describing where to allocate resources in order to optimize health and economic gains.

The MDGs focused on meeting the needs of the world’s poorest, and as this program comes to an end a new development agenda is forming. Health was strongly present in the MDG targets, and health will play a crucial, albeit less dominant, role in the 2030 Agenda for Sustainable Development. This program continues to grant significant attention to gender, and emphasizes women’s roles, both as individuals and as important contributors to economic and societal development. As a contribution to those discussions, this study attempts to gather and distill current evidence on the links among gender, health, and economic wellbeing.

Most diseases affect both sexes, but due to societal and biological factors, women and men are exposed to differing risks and barriers to health care. For example, the primary risk factor for lung disease in men is smoking, but for women it is household air pollution [10]. While women have lower mortality and live longer than men, with the gender gap in life expectancy rising from 4.8 to 5.7 years between 1970 and 2010 [11], young adult women have higher morbidity and bear a heavier burden of years of life lived with disability [12]. Women face persistent barriers in seeking health care and preventing disease, including gender discrimination, lack of education, and domestic violence [13]. Globally, great health inequities persist; data from the Global Burden of Disease Study (2013) show that the risk of maternal mortality is 19 times higher among women in developing countries than among their counterparts in developed countries [14]—among the greatest of all health disparities [13].

Because of these differences, we argue that the relationship between health and economic outcomes needs further analysis from a gender perspective. Previous reviews and reports have examined the economic spillovers associated with reproductive health [15–18]. In an effort to properly understand the full economic benefits of investments in health, we aim to extend this work by investigating the broader economic benefits of life-long investment in women’s health. We understand “investments in women’s health” to mean interventions that relate to women’s health. To also account for the negative effects of forgoing investment in women’s health, we have studied how the lack of interventions (as shown by poor women’s health) relates to economic development. We use fertility, intergenerational health spillover, education, productivity and savings as outcome variables when studying changes in women’s health (presented in Table 1).

**Table 1:** Outcome measures by pathways.

**Outcome measures**				
Fertility	Intergenerational Health Spillover	Education	Productivity	Savings
**Microeconomic level**				
Total fertility rate	Child survival	Enrollment in school	Income	Money
Change in fertility	Child wellbeing and behavior	Years of schooling	Purchasing power	Assets
Age at first birth/ teenage pregnancies	Anthropometry	Early drop out	Performance	
Birth spacing	Improved cognitive development	Performance in school		
	Life expectancy	Higher education		
	Adult health outcomes	Literacy		
	Nutrition			
	Intrauterine growth			
**Macroeconomic level**				

Gross domestic product/gross national product, gross domestic product/gross national product growth, income per capita, labor force participation, per capita income.

## Methods

To be able to understand the broader economic benefits of investing in women’s health, we reviewed and condensed the existing knowledge through a cross-disciplinary systematic review.

### Search Methods

We conducted a systematic review in accordance with the Preferred Reporting Items for Systematic Reviews and Meta-Analysis (PRISMA) guidelines [19]. The search algorithm is based on three main topics: women, health, and economics. We developed the search strategy to focus on women’s health in particular and did not include spillover effects on men’s health. The literature on investing in health documents known pathways through which health influences economic development: labor participation, productivity, savings, education, and fertility [7–9]. In addition, because a growing body of literature describes cross-generational health links, we included intergenerational health effects as a possible pathway. The search strategy was based on the authors’ knowledge of the literature, and some areas or pathways may have been missed. We deliberately set up a broad search algorithm; used a wide variety of search terms, including synonyms; and performed the search in health, economic, and development databases (PubMed/Medline; Embase; The Cochrane Library Economic Evaluations; EconLit; Web of Science/Science Citation Index Expanded; Cumulative Index to Nursing and Allied Health [CINAHL]; The National Bureau of Economic Research [NBER]; Latin and Caribbean Literature on Health Sciences database [LILACS]; Population, family planning, and related health issues [POPLINE]; and GenderWatch). We identified studies by searching 10 electronic databases, by snowball searching of references in key papers (pursuing the reference lists to identify additional papers), and by recommendations from colleagues. The search window was set from January 1, 1970, to April 1, 2013. The searches were performed in April 2013.

S1 Appendix provides a working protocol that details the search algorithms. As this is not a health care or social intervention review, the protocol was not registered in the Cochrane or Campbell Collaboration registries.

### Selection Process

All records captured by the search algorithms and obtained from references and colleague referrals were exported to an EndNote library. After eliminating duplicates, the two lead investigators (JI and KO) screened these records using title and abstract in order to perform an independent eligibility assessment in an unblinded, standardized manner [19]. Disagreements were resolved by consensus. Studies not meeting the eligibility criteria were excluded. After the first screening, the remaining records were extracted for full text review. When an article was not accessible through the Harvard University or University of Bergen libraries, we contacted the authors by email.

### Inclusion Criteria

We considered articles for inclusion if they were in English and reported on broader economic or developmental benefits resulting from improvements in women’s health throughout the life cycle (see Table 1 for detailed outcome measures). We included experimental studies (randomized controlled trials) and descriptive studies (including observational studies controlling for important confounders, and comprehensive qualitative studies).

### Exclusion Criteria

To ensure overall quality, articles considered to be methodologically simplistic (not experimental studies or descriptive studies) were excluded. The two main investigators assessed each study for potential selection bias (sampling bias or attrition bias) and reporting bias, and discussed the study design, reporting of results, and overall reliability. If the two main investigators agreed that there was a high chance of bias or low external validity in a study, it was excluded. We also excluded papers that reported on specific diseases not listed in the Global Burden of Disease 2010 cause list [10]. Studies published conducted prior to 1970 were excluded.

### Data Extraction

We developed a data extraction sheet based on recommended literature and similar reviews; this sheet was pilot-tested on a subset of the review articles. For each study, the primary authors independently gathered information on pathways to economic return, type of study, setting (country and income level of country), population studied, time period, aim of study, study design and methods, main result, risk of bias, quality of study, and strengths and limitations of the study. The primary authors discussed the information they had gathered from the studies and resolved disagreements about pathways and other characteristics. The authors reported on each study and its characteristics in a joint document (see S2 Appendix). Meta-analysis was not performed, as substantial differences exist between both outcome variables and methodologies in the studies included. Due to this diversity, we did not strictly enforce the PICO (Population, Intervention, Comparison and Outcome) approach.

As the aim of the study was to investigate the broader economic benefits of investing in women’s health, we chose to report the review findings based on the pathways in Table 1 and the extraction table (S2 Appendix). The authors discussed any disagreement about the understanding of the papers or pathways and consensus was reached concerning each study.

## Results

This review finds that women’s health is tied to economic development.

### Results of Review

**Fig 1:**
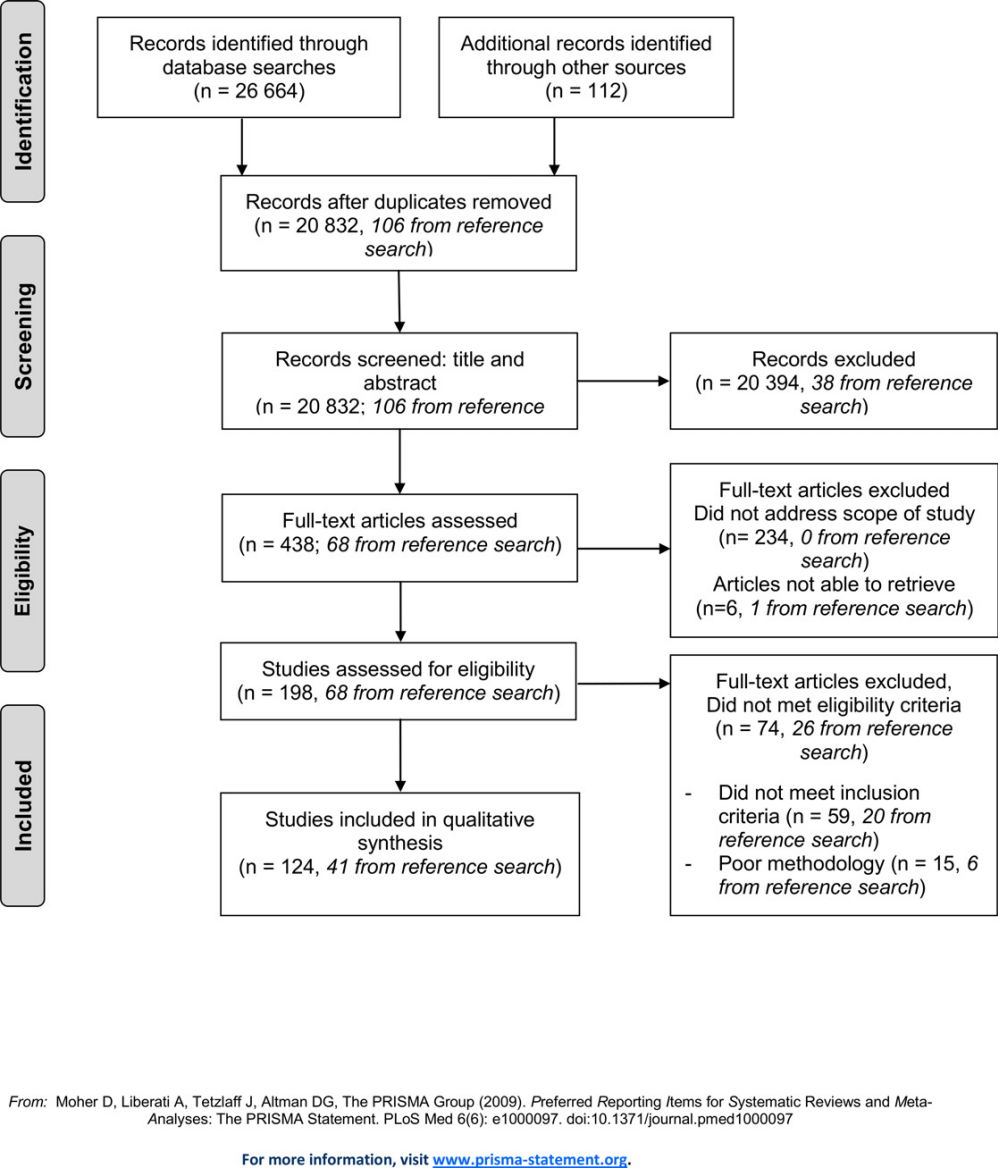
The Data Extraction Process.

The search algorithms captured 26,664 records, and references and colleague referrals yielded 112 additional records. We excluded 5,944 duplicates. The two lead investigators independently screened 20,834 abstracts, extracting 438 records for full text review. For the six articles that were not accessible through the Harvard University or University of Bergen libraries, we contacted the authors by email but ultimately were unable to secure the publications. The final review reflects the inclusion of 124 articles (Fig 1), with 41 originating from the reference search. The most common reasons for excluding studies from the review were that the studies did not address the scope of this study, or did not meet the study design inclusion criteria (i.e. were not experimental or observational studies). The most common reasons for excluding studies from the final review were that they did not meet the inclusion criteria (59) or poor methodology (15), e.g. failure to control for important confounders. The potential bias and validity concerns for each study are reported in S2 Appendix.

Table 2 summarizes the included studies based on pathways. The majority of the studies examine intergenerational spillovers, and some describe how women’s health relates to more than one pathway.

**Table 2:** Pathways: Women’s health and economic development.

Fertility	Intergenerational Health Spillover	Education	Productivity	Savings
13	95	35	32	7

### Fertility and Women’s Health: The Demographic Dividend

Bloom, Canning, and Sevilla describe how declines in fertility can boost economic growth through a phenomenon they label the demographic dividend [20]. When a given population transitions from high to low rates of mortality and fertility, a bulge occurs in the population age structure as mortality rates tend to drop before fertility rates. Coupled with a sound policy environment and labor opportunities, this “boom” generation can boost a country’s economy when it reaches working age through increased productivity and savings, and reduced dependency ratios [20]. Quantifying the economic gains from reduced fertility is difficult due to complex development processes are complex, but Ashraf et al. make an attempt using a demographic simulation model [21]. They estimate that the effect of an exogenous fertility reduction in a UN low-fertility population projection in Nigeria would raise per capita income by 5.6% and 11.9% over a horizon of 20 and 50 years, respectively [21]. Recent work by Bloom and colleagues suggests that Kenya, Nigeria, and Senegal could each increase per capita income by 8–13% over the baseline scenario, wherein none of the unmet need for contraception is met, by meeting one third of the unmet need for family planning. This strategy would capture the demographic dividend; if each country meets all unmet need for contraception, per capita income could increase by 31–65% [22].

Some U.S. studies show how women’s control over their own fertility, through access to contraception and family planning, is also crucial for women’s opportunities in higher education and employment. Goldin and Katz found the availability of oral contraception in the U.S. to be associated with increased age at marriage and a greater number of women pursuing higher education and professional careers [23]. Family planning programs can influence national income directly through long-term effects on household finances and increased access to education. Children conceived in areas with greater access to family planning were 2–7% more likely to attain higher education and to live in higher-earning households [24].

Family planning enables women to have better-planned pregnancies and healthier babies. Access to birth control can reduce the likelihood of young maternal age at first birth (i.e. before age 22), increase the number of women participating in paid labor (by 8% among 26–30 year olds), raise the number of annual hours worked by women [25], and accelerate the reduction in birth rates by up to 40% [26]. Jamieson and Buescher found that women in the U.S. who used family planning services two years before they got pregnant were less likely to deliver low birth weight babies (though the findings were only statistically significant for African Americans) [27].

Abortion has been studied as a proxy for fertility control, and abortion legislation and access have been shown to affect education and productivity. The abortion ban implemented in Romania in 1967 is associated with worsened educational and labor market achievements for children born after the ban, after controlling for observable background variables [28]. Ananat et al. found that greater access to abortion in the U.S. is associated with higher rates of college graduation, lower rates of single parenthood, and lower odds of welfare receipt. [29]. Access to legalized abortion provides working women the option to avoid unwanted pregnancies and maintain their employment status; difference-in-difference analysis of the Roe vs. Wade case in the U.S. suggests that legal access to abortion services increased labor force participation rates, particularly among African American women [30]. Using abortion legislation as an instrument for fertility control in a cross-country study, Bloom et al. found a negative effect of high total fertility rates on female labor force supply. They estimate that each birth on average reduces a reproductive woman’s lifetime labor supply by almost two years [31]. Removing abortion restrictions can reduce fertility and allow women to contribute more to the labor force.

Improved reproductive health services are also shown shown to benefit the societies of low- and middle-income countries (LMICs). Results from Matlab, Bangladesh, show that the drop in fertility rates in areas with improved access to reproductive health services was associated with increases in women’s earnings and assets [32] and decreases in fertility [33]. Child survival, schooling, and mother and daughter body mass indexes (BMI) all improved in project villages, and physical household assets were 25% greater than in non-program intervention areas. Interestingly, even though this program only targeted women of reproductive age and their children, it also improved the health of the elderly women living in the households, indicating a positive spillover effect [33]. A similar experimental study in Ghana delivered enhanced reproductive health services to 83,000 randomly selected women between 1995 and 2011, and documented an initial decrease in fertility. However, in the last decade of the study, fertility stagnated or increased, possibly because the project neglected social mobilization and community ownership while focusing on other areas [34]. The study did not examine how the project affected society beyond the initial decrease in fertility. However, Miller found that contraception explains less than 10% of fertility reduction during Colombia’s demographic transition between 1964 and 1993, concluding that fertility is also influenced by many other factors[35].

### Intergenerational Health Spillovers and Women’s Health: Maternal Health and Generational Dividends

Over her lifetime, a woman’s health has profound consequences for subsequent generations. Poor maternal health is associated with diminished child health, with implications for birth weight, neonatal survival, cognitive development, child behavior, school performance, and adult health and productivity [36–41]. Victora et al. conducted an important review on associations between maternal and child undernutrition and adult health and human capital based on data from five LMIC cohorts [37]. The authors conclude that ill health in childhood leads to long-term damage and may affect future generations; its prevention is therefore crucial to tapping “important health, educational, and economic benefits” [37].

#### Nutrition

In 1995, Barker wrote that “coronary heart disease is associated with specific patterns of disproportionate fetal growth that result from fetal undernutrition in middle to late gestation”—later termed the fetal origins hypothesis [42]. This commenced a new field of research examining how events before birth influence future health [43]. Famine studies provide natural experiments to evaluate the long-term effects of fetal undernutrition, and Dutch and Chinese famine studies show that poor fetal growth is associated with higher risk of poor nutrition in utero, stunting, hyperglycemia, type 2 diabetes, cardiovascular disease, and premature mortality [44–48]. Studies from China also find that fetal exposure to acute maternal malnutrition negatively impacted literacy, income, labor market status, and marriage market outcomes [49, 50]. A study of the Greek famine finds a significant reduction in years of schooling for those exposed to famine in utero [51]. Hult et al. investigated the long-term health consequences of the Biafran famine in Nigeria and found that fetal and infant undernutrition is associated with significantly increased risk of hypertension and impaired glucose tolerance in 40-year old Nigerians [52].

Micronutrient deficiencies, anemia, stunting and low weight, height, and BMI in mothers all increase the risk of intrauterine growth restriction, low birth weight, poor newborn health, stunting, and wasting in their children [53–64]. Much of this knowledge derives from a large, longitudinal supplementary feeding trial in two pairs of two Guatemalan villages, conducted between 1969 and 1977 by the Institute of Nutrition of Central America and Panama (INCAP) [65]. One village in each pair was randomly selected to receive a high-protein, high-energy supplement, Atole, while the other village received a no-protein, low-energy supplement, Fresco. All pregnant or lactating women and all children from birth to seven years of age were included, exposing all children to either of the supplements at different ages and for different periods of time [66]. Four follow-up studies on these mothers and children demonstrated that nutritional interventions in children under three years of age have a broad range of long-term effects. Child length gain during the first three years of life was greater in the Atole villages, and in follow-up studies these adolescents were taller, weighed more, and had greater lean body mass than subjects from Fresco villages [67, 68]. Further, the nutritional supplementation of girls in early childhood was found to have significant positive effects on the body size of their later offspring [69].

Maternal malnutrition perceived as overnutrition can also be harmful. Cresswell et al. show that maternal obesity increases the risk of neonatal death in 27 African countries [70]. When looking at stunting and BMI among children in South Africa and Brazil, respectively, children of overweight mothers face a significantly greater risk of being overweight. The association stayed significant after controlling for various socioeconomic factors [71, 72].

Determining whether the effects of improved maternal nutrition on child health result from better intrauterine conditions or improved nutrition as a young child is difficult. The previously described famine studies imply that the intrauterine environment is of great importance, but the literature is not conclusive. Subramanian et al. found that the association between childhood undernutrition and either paternal BMI or maternal BMI is of the same magnitude, indicating that intrauterine conditions may not directly determine nutritional status of offspring [73]. Even so, most literature points toward poor maternal health as being the greater risk factor for poor newborn and infant health, increasing the risk of child stunting and reduced cognitive development and affecting later productivity [74–77].

**Fig 2:**
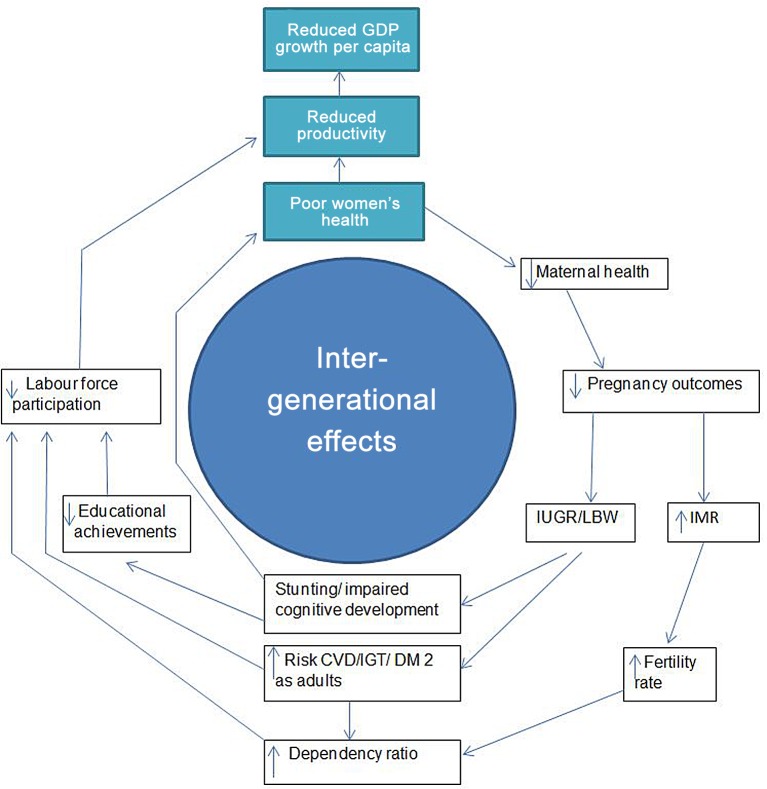
The virtuous spiral of intergenerational health effects. Fig abbreviations: IMR: infant mortality rate, IUGR: Intrauterine growth restriction, LBW: low birth weight, CVD: cardiovascular disease, IGT: impaired glucose tolerance, DM2: diabetes mellitus type 2.

Improved maternal nutrition may offer the dual benefits of benefiting economic development through improved human capital accumulation, and improving the health of future generations (Fig 2). One example is an iodine intervention study from Tanzania, which found that children of mothers living in areas with iodine supplementation programs attained an estimated 0.35–0.56 years of additional schooling, compared with children living in areas without supplement programs. The effect appeared to be larger for girls, consistent with laboratory evidence indicating greater sensitivity in female fetuses to maternal iodine deficiency [78]. In line with these findings, a study from the Chinese famine (1959–1961) reaffirms that exposure to nutritional adversity in early life may have larger long-term consequences for women than for men, manifested as increased likelihood of disability and illiteracy. However, the authors highlight that results can be confounded by factors such as excess male mortality and cultural dynamics such as son preference [79].

Substantial literature indicates that lower maternal height, as a proxy for maternal health in early life and childhood, is associated with worse child health and nutrition [38, 80–83], particularly for shorter women born in poorer socioeconomic conditions [84]. A pooled analysis using data from of five cohorts found that height of mothers is associated with their children’s birth weight, height, and conditional height based on previous growth [81]. Some studies show similar effects of paternal height, but the association is not as strong [80]. Researchers found significant relationships between parental and child health during the first three years of life by using various health criteria, including anthropometric measures, information on health disorders, and “self-rated” health reports from a German panel dataset, and controlling for parental income, education, and family composition. Cross-sectional findings from German mothers and children suggest that parental health tends to be transmitted to the child via the mother [85].

The health of a mother can affect the health of subsequent generations. A U.S. study found that grandmother’s stature and mother’s birth weight were the strongest predictors of the mother’s stature, which in turn has implications for the birth weight of their children [86]. Currie and Moretti found that low maternal birth weight is associated with low birth weight among offspring, even among mothers who are sisters and therefore exposed to similar genetic material and environment [87]. Interestingly, the intergenerational transmission of low birth weight is stronger for mothers in high-poverty zip codes in the western U.S. and is also associated with subsequently lower socioeconomic status [87]. Several studies have shown that stunted children have lower school performance and are less productive as adults than nonstunted children in adulthood [66, 88, 89], offering strong arguments for improving women’s nutritional status as a tool to break the cycle of poverty.

#### Maternal Orphans and Health

The presence of a mother is an important protective factor for child survival; studies in LMICs have found an increased mortality risk for children following a mother’s death [90–92]. A cohort study from Matlab, Bangladesh, showed that the cumulative probability of children surviving to their tenth birthday was 24% for children whose mothers died, while the effect of paternal death before child survival was negligible [92]. Studies from South Africa, Kenya and Indonesia indicate that maternal orphans are less likely to be enrolled in school than paternal orphans, but the impact seems to differ between countries [93–96]. A Tanzanian study did not find as large differences in schooling attainment between maternal and paternal orphans, but did find that maternal orphans have 15% lower height [97].]. Maternal orphanhood is also associated with increased sexual risk behaviors, prevalence of sexually transmitted infections, and poor mental health in children [98–102].

#### Maternal Mental Health

Maternal mental health and disorders affect the production of future human capital through increased risk of poor child health, including neonatal mortality, low birth weight, later stunting, and diarrheal disease [103–113]. However, the evidence is mixed and seems to depend on setting and study design. Findings from India [104], Pakistan [108–110], Brazil [106], and Bangladesh [111, 112] suggest negative effects of maternal mental disorders on child health, but have not been replicated in studies from African countries [113–118], Taiwan [119], the U.S., or Brazil [104, 120–122]. In a systematic review, Surkan et al. find varying results across studies, but their meta-analysis concludes that maternal depression was associated with early childhood underweight and stunting. However, this conclusion was based upon studies that differed in study designs, was conducted in a wide range of locations and included children of different ages. [123].

Although the evidence is mixed when it comes to common mental disorders, it is clearer when looking at severe mental disorders, like psychosis and schizophrenia [118]. Offspring of mothers with these diseases have higher morbidity and mortality, suggesting a possible dose-dependent response to accumulation of risk that affects child development [124].

Studies from high-income countries show that maternal mental illness has negative effects for offspring health, behavior, and psychosocial functioning [125–128]. The extent to which societies and cultures have different coping mechanisms and social support to look after the child when a mother (or father) falls ill will probably affect how much maternal mental health affects child health and wellbeing. Interestingly, a U.S. study shows that high paternal involvement does not compensate for the mother’s health problems [129].

#### Age of Childbearing

Maternal age seems to affect child health outcomes, wherein the youngest and oldest age groups have poorer outcomes. Gibbs et al. conclude in their meta-analysis that young maternal age increases the risks of low birth weight and preterm birth, though the overall evidence of the effect is moderate [130]. A study looking at adverse maternal and perinatal outcomes in 850,000 Latin American women found the risk of dying from pregnancy-related causes to be four times higher for adolescents under 16 years old than for women in their early twenties [131]. A South African study investigated the social and economic consequences of teenage motherhood and found large educational attainment deficits in teen mothers, with earlier births associated with greater deficits [132]. Studies indicate that household characteristics may explain poorer pregnancy outcomes for young mothers, but the degree of impact differs between settings [133–134].

Studies from Canada and South Africa looked at long-term consequences for children born to teenage mothers, showing negative impacts on educational achievement, life satisfaction, and personal income later in life [135, 136]. In a 55-country study based on data from the Demographic and Health Survey (DHS), Finlay at al. find that women who have their first birth between the ages of 27 and 29 have the lowest risk of poor child health outcomes. They note that both biological and social mechanisms determine why children of young mothers have poorer health outcomes [137]. The factors that co-vary with both age and child outcomes differ by countries and by urban/rural location within a country and require further study.

In addition to being a health risk for both the mother and the child, teenage childbearing imposes a sizable economic burden in terms of potential productivity gains [138]. If young girls had delayed pregnancy, attained higher education, and been employed the opportunity costs can be high; from 1% (China) to 30% (Uganda) of gross domestic product (GDP) [138].

Similar difficulties appear in the literature surrounding the consequences of birth spacing. A meta-analysis from 2006 examines the association between birth spacing and the relative risk of low birth weight, being born small for gestational age, and preterm birth. The study finds that inter-pregnancy intervals shorter than 6 months, between 6 to 17 months, and longer than 59 months are associated with significantly greater risk of adverse pregnancy outcomes [139]. A long-term study using data from Matlab, Bangladesh, finds that the risk of infant mortality is significantly increased when inter-pregnancy intervals are short [140].

### Education and Women’s Health

The effect of improved women’s health on education works primarily through fertility and intergenerational health channels, as discussed previously. However, looking at school achievement and economic productivity, the Institute of Nutrition of Central America and Panama (INCAP) studies found that nutritional supplementation increased years of schooling significantly (by 1.2 grades) for women, but not for men [141]. Both men and women exposed to Atole, the nutritious supplement, within the first two years of life showed improved comprehension and intelligence scores, independent of schooling [142]. Another effect of Atole exposure was a 46% increase in average wages for men, but not for women; one explanation for the difference is that fewer women than men engaged in income-generating activities [143]. In Sri Lanka, Jayachandran and Lleras-Muney find a dramatic reduction in maternal mortality risk (70%) between 1946 and 1953 to be associated with increased investments in girls’ education [144]. Using Chinese data, Sun et al. estimate that a 0.1% decline in the maternal mortality rate would reduce the female illiteracy rate by 6.1–12.8% [145]. A study from rural Pakistan indicates that improved nutritional status increased school enrollment for children, in particular among girls, thereby reducing the gender gap in school enrollment [146].

### Productivity and Women’s Health: Labor Force Participation and Economic Return

Poor health among women has not only generational repercussions, but also implications for contemporaneous labor force participation, productivity, earnings, family income, and economic wellbeing. When looking at labor force participation, a longitudinal study from Australia found that having diabetes, high blood pressure, or psychiatric conditions decreased the likelihood of women’s employment [147]. Globally, only 52% of the world’s adult women participate in the labor market, 26% lower than men. Childbearing, illness, and complications from pregnancy and delivery predispose women for absence from work. The prevalence of traditional gender roles also prevents women from joining the labor market, as they are often allocated the time-consuming task of child-rearing [148]. Interventions that improve safety in pregnancy and delivery, together with fertility reductions, are shown to increase female labor force participation. Albanesi and Olivetti find that reduced maternal mortality and access to high-quality infant formula in the U.S. enabled women to engage in both paid work and in the duties of motherhood, resulting in a 52% rise in work participation among women aged 23–33 years between 1920 and 1970 [149]. A modeling study from the WHO African Region (AFRO) estimates that reducing maternal deaths could result in a significant economic return: each maternal death decreases per capita GDP by US$0.36 per year [150].

Improved health, in particular improved nutrition, boosts economic returns through increased productivity. In China, a group of researchers estimated that the reduction in goiters from 1992–2001 increased the net present value of future economic productivity by 142 billion yuan (roughly US$23 billion at the June 2014 exchange rate, estimates by authors of this study) and that a continued reduction of the goiter rate to 5% over the next 10 years would result in future, additional productivity gains with a value of 40 billion yuan. The reduction in prevalence of child stunting from 32.7% to 14.4% is estimated to have resulted in economic productivity gains with a net value of an additional 101 billion yuan [151]. Because micronutrient deficiencies constitute a higher burden in women than in men, in particular iron deficiency anemia among women of reproductive age [10], improving these deficiencies can have a major positive impact on both the productivity of the targeted generation and on the cognitive development and later productivity of generations to come.

Health and productivity are positively related for both men and women. Nevertheless, female farmers have, on average, 20–30% lower productivity than male farmers, and female entrepreneurs also exhibit lower productivity [148]. The factors influencing female and male productivity are complex and varied, and include differences in education, time allocation between domestic and income-generating work, and access to the formal labor market [148]. One important determinant might be poor health and the higher burden of nutritional deficiencies in women than in men [152]. An intervention study in India looked at the effects of multi-micronutrient supplements on female tea pickers and found a significant increase in labor productivity [153]. Anemic adolescent girls in India have poorer results in memory tests, reduced work capacity, and lower cognitive function than their non-anemic counterparts [154].

A study from Colombia suggests that a one-centimeter increase in mean height would lead to a 12% increase in earnings for men but only a 4.7% increase for women [155]. Studies of HIV treatment indicate that antiretroviral treatment (ART) can lead to improvements in worker productivity, but the literature indicates different impacts between men and women [156–158]. A micro-level study shows an intergenerational effect: adult deaths increase the likelihood of labor force participation among male and female adolescents, with a greater negative effect on females that leads to decreased female school enrollment [156].

ART has led to massive health gains for HIV patients, and these health benefits have the potential to significantly improve economic wellbeing [157], but whether ART can reverse the declines in labor productivity that typically follow severe disease is still debatable. This is a critical inquiry because labor is the central productive asset of the poor in many LMICs. A study from Western Kenya found a 20% increased likelihood of participation in the labor force six months after ART initiation along with a 35% (7.9 hours) increase in weekly hours worked [158]. Treatment effects for women were primarily on the extensive margin—indicating that they either leave or enter the labor force—while the treatment effects for men are on the intensive margin, meaning they can work more hours. Much of this gender difference is probably due to differences in domestic time allocation, which is a determinant for children’s school enrollment and continuation [158]. In another study of tea pickers, also from Western Kenya, Larson et al. confirm that women tend to benefit from ART on the extensive margin [159].

Measuring formal labor might therefore not capture the true productivity effects of ART uptake. Cultural and social gender norms influence how poorer health might affect productivity among women and men differently [148]. In particular, more traditional gender roles correlate negatively with labor force participation, as the impact of ART for women tends to be on the extensive margin. However, the possible importance of this should not be underestimated, especially when examining time allocation for domestic chores. Providing ART and improving the health of mothers improves children’s educational attainment, in particular that of girls, because they do not have to drop out of school to assume the burden of domestic work. Despite the challenges of capturing the whole picture, increasing access to health services for women has been shown effective in improving the health and thereby productivity of the population.

### Savings and Women’s Health

Little evidence exists on the role of women’s health on savings, but maternal complications and death can negatively impact household finances. Out-of-pocket expenditures often cover most health care costs in poor households [91, 160, 161], and the consequences of women falling sick include lost productivity during illness, increased debt, and reduced savings, pushing poor or already marginal households further into poverty [91, 161, 162].

## Discussion

Most studies included are cross-sectional correlational studies describing associations, with only a limited number of long-term and experimental studies published. The cross-sectional studies have in particular identified important intergenerational associations, but further studies are needed to understand potential causal relationships. Previous literature reviews have focused on particular aspects of economic development [15–18], while this review takes a life span approach and has selected studies from a holistic view, including women’s reproductive, sexual, physical and mental health. While the majority of the literature defines women’s health as limited to reproductive health, we identify how women’s overall health relates to economic development. We attempt a broader understanding of women’s health that expands the economic importance of women beyond reproduction. Our review captures the relevance of women’s mental health and acknowledges intergenerational effects as economic outcomes.

Our study used a comprehensive search strategy drawing from ten databases in different academic fields. Each paper was assessed for bias, as reported in S2 Appendix. Nevertheless, there may remain reporting biases in the original literature on women’s health and economic development [19]. Our restriction to English-language publications may have excluded important literature in other languages. Thorough application of well-defined inclusion and exclusion criteria, independent screening by two authors, and use of the tested extraction table increase the reliability of our results. While other researchers might interpret some of the study results differently, we argue that our rigorous methodology would support another team achieving similar results when using the same approach.

The cross-disciplinary nature of our review is one of its strengths, but we were surprised to see a strict divide and lack of cross-referencing between the health and the economic literatures. As we believe cross-disciplinary work can promote more efficient use of existing knowledge, disciplinary silos should be avoided. Further studies of the benefits of specific health investments should consider literature from the economic, development, gender, and health fields,. We hope that this literature review can be a starting point for such cross-disciplinary endeavors.

Women’s health is linked to our understandings of women from biological and societal perspectives, by focusing on “sex” or on “gender.” The majority of the literature included examined women’s health from a biological perspective, with little emphasis on the social constructs that shape our understanding of gender roles [163].

Most studies reviewed describe the costs of poor health in women—in particular the costs of poor maternal health—while a minority of the studies describe how health investments lead to economic benefits. New studies should aim for long-term and experimental designs to better understand the potential causal pathways. However, the associations found in this study are important for health policy today, as they indicate important linkages between women’s health and economic outcomes.

In general, evidence providing economic justification for investments in women’s health tend to focus on reproductive health, investigating the implications of women’s reproductive rather than productive roles [15–18]. Looking at the limited number of studies comparing productivity by gender, healthy men appear to be more productive than healthy women, but the impact of ill health affects both men and women. Gender roles vary in different countries, and men’s productivity is most highly valued in those societies where males are the traditional breadwinners. But in a long-term consideration of the impacts on fertility, health, and education benefits for future generations, women’s health and accompanying productivity must receive more attention.

Most of the literature on the broader benefits of investing in women’s health investigates intergenerational effects, productivity, and education. Most studies of the effect of health on productivity try to quantify an increase in manual labor input or output in traditional societies with larger gender inequities. Further study is needed in settings with smaller inequities and higher opportunity costs for women to be outside the formal labor force. The lack of studies about the relationship between women’s health and savings indicates a need for further investigation. Research from townships in South Africa shows that grandmothers tend to save relatively more of their retirement pensions than grandfathers and invest more of the savings in their granddaughters [164]. Some hypothesize that women have a greater propensity to save than men and that those savings are distributed to the family [165–169].

Good health among women is important for child development and the production of future human capital. Benefits from reproductive health investment, including access to contraceptives and abortion, can yield high gains for the women themselves, their families, and their societies. When it comes to the direct contribution of a woman’s health to economic development in her own generation, the literature is suggestive, but not decisive, and highly contextual. As the conclusions of this review are based on studies in low-, middle-, and high-income countries, this strengthens the generalizability of the review. However, contextualized analysis that accounts for cultural factors is needed, as the status of women differs among and within countries [170].

Our study describes spillovers from investments in women’s health that are not covered in current cost-effectiveness analyses. By using cost-benefit analysis for women’s health and other health outcomes, we may better capture the true economic benefits of health improvements and interventions. Though this study does not capture the costs and benefits of all interventions with regard to women’s health or provide quantitative cost-benefit analysis for policy makers, our findings point toward investments in the health of girls and women as a potent development opportunity for current and future generations.

## Conclusion

Our multidisciplinary and robust systematic review of existing literature on women’s health and economic development supports four main conclusions. First, healthier women contribute to better-educated and more productive societies. Second, ensuring women’s control over their own fertility can boost the pace of economic growth and development. Third, maternal health is crucial to the health and economic wellbeing of subsequent generations through intergenerational spillovers. Fourth, further study is needed on women’s health and household and societal productivity.

In the short-term perspective, men are often the family breadwinners, and their health is therefore perceived as vital for family welfare. However, in the long-term perspective, women’s health is fundamental for the health and development of future generations [171].

Previous research has focused on the importance of women’s health based on human rights and social equity theories. This study demonstrates that women’s health is tied to productivity: how nations develop and perform depends upon how the country educates and provides opportunities for its women. The studies reviewed herein are generally consistent with the view that strong links exist among deliberate family planning; the health of the mother before, during, and after birth; and the health and productivity of the next generation, a self-perpetuating cycle of societal development. Mothers give birth to the workforce of the next generation, and physically and emotionally healthy workers tend to be more productive workers. How health affects the productivity of men and women differently needs further attention, which a growing body of literature reveals is a worthy pursuit. These results demonstrate the necessity and efficacy of investment in initiatives that address women’s health. Societies that invest in women’s health will likely have better overall population health and will remain more productive for generations to come.

## Supporting Information

S1 Appendix Review Protocol: Economic benefits of investing in women’s health: A systematic review. (DOCX)

S2 Appendix Extraction table: Articles in included in the systematic review. (XLSX)

S3 Appendix PRIMA Checklist: Economic Benefits of Investing in Women’s Health: A systematic Review. (DOC)
