# Supplementary material for: Economic Benefits of Investing in Women’s Health: A Systematic Review
Source: PLoS One. 2016 Mar 30;11(3):e0150120. doi: 10.1371/journal.pone.0150120 (PMC4814064; doi:10.1371/journal.pone.0150120)
Supplement: S1 Appendix — (DOCX) [file pone.0150120.s001.docx]

# S1Appendix

| **Main title** | Economic benefits of investing in women’s health: A systematic review |
| --- | --- |
| **Sub title** | Systematic review of the literature on the effect of women’s health on  individual and family/household income and economic growth |
| **Institution** | Harvard School of Public Health  Department of Global Health and Population |
| **Review Group** | Johanne Helene Iversen (JHI), principal investigator (PI)  Kristine Husøy Onarheim (KHO), principal investigator (PI)  David Bloom (DB), co-author/advisor/investigator |
| **Contact details** | kristine.onarheim[at]uib.no or johanne.h.iversen[at]gmail.com |
| **Month/year of publications** | 2014 |
| **Conflict of interest** | No conflict of interest |
| **Acknowledgments** | This study was funded by the Norwegian Agency for Development Cooperation (NORAD) |
| **Reference number** | Protocol not registered |

# Review Protocol: Economic benefits of investing in women’s health: A systematic review

Kristine Husøy Onarheim, Johanne Helene Iversen, David E. Bloom

# Content

[Review Protocol: Economic benefits of investing in women’s health: A systematic review 1](#_Toc419018135)

[Background 3](#_Toc419018136)

[Rationale 4](#_Toc419018137)

[Review Objectives 5](#_Toc419018138)

[Methods 5](#_Toc419018139)

[Sources of Information 5](#_Toc419018140)

[Search Terms 6](#_Toc419018141)

[PubMed Search Algorithm 7](#_Toc419018142)

[Eligibility Criteria 8](#_Toc419018143)

[Data Extraction 9](#_Toc419018144)

[Role of Reviewers 10](#_Toc419018145)

[Data Synthesis 11](#_Toc419018146)

[Assessment of Bias Risk 11](#_Toc419018147)

[Final Search Strategy by Databases 11](#_Toc419018148)

# Background

After the World Bank’s 1993 *World Development Report*: *Investing in Health* and the 2001 report of the WHO’s Commission on Macroeconomics and Health, researchers have studied the various pathways through which health can lead to economic development:

- **First,** a healthier workforce is a more productive workforce: it has more energy, better mental health, and lower rates of absenteeism.
- **Second**, healthier children do better while they are in school and tend to have better records of school attendance; they also tend to stay in school longer since longer-lived and healthier populations have more incentive to invest in education.
- **Third,** healthy populations have higher savings rates, as people save more in anticipation of longer periods of retirement.
- **Fourth,** healthy populations tend to attract more foreign direct investment.
- **Fifth**, improvements in child survival trigger a decline in fertility, which allows countries to escape the burden of youth dependency, wherein too large of a percentage of the population is not old enough to join the productive labor force, and to enjoy rapid economic growth in the form of a demographic dividend.

The past 15 years have seen extensive research conducted on the overall healthier-means-wealthier proposition and the individual pathways noted above. This research supports the breakthrough finding that population health is an exceedingly robust and powerful driver of economic growth and poverty alleviation, and that all five pathways are operative.

Globally, the actual health of women falls short of its potential, primarily due to structural and avoidable obstacles like gender discrimination, lack of education, domestic violence, lack of access to family planning and reproductive health services, and the human trafficking of women and girls. In addition to the disturbing ethical and human rights implications of the deficit in women’s health, there are likely economic consequences related to (1) lowered productivity and labor income; (2) impeded school enrollment, attendance, and learning; (3) diminished household savings (sometimes forcing many households to spend down their savings to pay medical bills); and (4) prevention of women and their partners from reducing their fertility.

One more reason why most of these effects are even more consequential from a macro standpoint is because women are central to the well-being of others in their families and communities. If women are unhealthy, the health, nutrition, education, and emotional well-being of others around them also tend to be adversely affected. In other words, ill health can wipe out the sizable beneficial spillovers that women confer on their families and communities.

The overall goal of this project is to outline, study, and test the linkages between women’s health and economic well-being and growth and to inform the efforts of the Norwegian Agency for Development Cooperation’s global health agenda, the World Bank’s work in this area, and the Lancet Commission on Investing in Health.

### Rationale

Robust evidence links population health to economic development. However, within this body of research, the specific role of women’s health as a driver of economic development is poorly understood. To inform global health investment going forward, the specific contribution of women—both in terms of their own health status and their role in promoting the health of family and community members—needs to be better understood at a micro- and macroeconomic level.

### Review Objectives

We aim to systematically review health, gender, and economic literature for studies that have investigated the impact of women’s health on economic outcomes, both at the micro- (household/family) and macroeconomic (country) levels (economic growth).

Our hypothesis is that women`s health has a relatively large effect on individual and household economic well-being and on the pace of economic growth. In part, this occurs because improvements in women’s health confer a wide range of beneficial spillover effects on families, communities, and generations to come.

# Methods

The method is developed according to the Preferred Reporting Items for Systematic Reviews and Meta-Analysis (PRISMA) guidelines.

### Sources of Information

We will search the health, gender, and economics literature for relevant studies and will use the following electronic databases:

- PubMed/Medline
- Embase
- The Cochrane Library Economic Evaluations
- EconLit
- Web of Science/Science Citation Index Expanded
- Cumulative Index to Nursing and Allied Health (CINAHL)
- The National Bureau of Economic Research (NBER)
- Latin and Caribbean Literature on health sciences database (LILACS)
- POPLINE (Population, family planning, and related health issues)
- GenderWatch

In addition to searching the listed databases, we will identify articles through snowball searching of references in key papers and recommendations from colleagues.

### Search Terms

We will develop a search algorithm based on three main topics: women, health, and economics. The algorithm will focus on women’s health in particular and will not include spillover effects of improvements in men’s health. The literature on investing in health describes known pathways through which health is documented to influence economic development: productivity, savings, education, and fertility. In addition, we include intergenerational health effects as a possible pathway, as a growing body of literature describes cross-generational health links. The search strategy is based on the authors’ knowledge of the literature and areas or pathways that might have been missed in the search. We will deliberately set up a broad search algorithm and include a wide variety of search terms, including synonyms.

The initial search algorithm was developed with inspiration from the review “The Economic Benefits of Investing in Reproductive, Maternal, Newborn and Child Health: A Systematic Literature Review,” conducted by researchers from Lund University in 2011, and with help from Carol Ann Mita, librarian at Francis A. Countway Library of Medicine in Boston, Massachusetts. The search algorithm was developed for PubMed, and minor editions have been made for the other databases.

### PubMed Search Algorithm

("Economic Development"[Mesh] OR "Work"[Mesh:NoExp] OR "Employment"[Mesh:NoExp] OR "Salaries and Fringe Benefits"[Mesh:NoExp] OR "Income"[Mesh:NoExp] OR "Poverty/economics"[Mesh] OR "Poverty/prevention and control"[Mesh] OR earnings[tiab] OR economic benefit[tiab] OR economic consequences[tiab] OR economic contribution[tiab] OR economic development[tiab] OR economic growth[tiab] OR economic impact[tiab] OR economic improvement*[tiab] OR economic investment*[tiab] OR economic productivity[tiab] OR economic returns[tiab] OR economic situation[tiab] OR economic status[tiab] OR economic well being[tiab] OR formal labor[tiab] OR gross domestic product[tiab] OR gross national product[tiab] OR household consumption[tiab] OR income[tiab] OR incomes[tiab] OR informal labor[tiab] OR informal sector[tiab] OR labor force[tiab] OR labour force[tiab] OR labor market*[tiab] OR labour market*[tiab] OR labor participation[tiab] OR labour participation[tiab] OR macroeconomic*[tiab] OR macroeconomic[tiab] OR microcredit[tiab] OR micro credit[tiab] OR microeconomic*[tiab] OR micro economic[tiab] OR paid labor[tiab] OR paid labour[tiab] OR poverty alleviation[tiab] OR poverty reduction[tiab] OR reduce poverty[tiab] OR salary[tiab] OR salaries[tiab] OR savings[tiab] OR wages[tiab]) AND ("Mortality"[Mesh:NoExp] OR "Morbidity"[Mesh:noexp] OR "Child Mortality"[Mesh] OR "Infant Mortality"[Mesh] OR "Infanticide"[Mesh] OR "Malnutrition"[Mesh] OR "Maternal Mortality"[Mesh] OR "Perinatal Mortality"[Mesh] OR "Reproductive Health"[Mesh] OR "Women's Health"[Mesh] OR adolescent health[tiab] OR burden of disease[tiab] OR child health[tiab] OR children's health[tiab] OR child mortality[tiab] OR disease burden[tiab] OR female morbidity[tiab] OR female mortality[tiab] OR infant mortality[tiab] OR infanticide[tiab] OR malnutrition[tiab] OR maternal health[tiab] OR maternal morbidity[tiab] OR maternal mortality[tiab] OR mortality decline*[tiab] OR neonatal mortality[tiab] OR perinatal mortality[tiab] OR reproductive health[tiab] OR ((abortion[tiab] OR abortions[tiab]) AND (sex selective[tiab] OR sex biased[tiab] OR sex preference* [tiab] OR son preference[tiab] OR sex selection[tiab] OR sex selected[tiab] OR missing girls[tiab] OR gender imbalance[tiab] OR female feticide[tiab] OR female fetus*[tiab])) OR sexual health[tiab] OR undernutrition[tiab]) AND ("Female"[Mesh] OR "Women's Health"[Mesh] OR adolescent girls[tiab] OR adult women[tiab] OR elderly women[tiab] OR gender[tiab] OR older women[tiab] OR women's health[tiab] OR young women[tiab] OR maternal[tiab] OR female[tiab] OR female’s[tiab] OR females[tiab] OR women[tiab] OR women’s[tiab])

See the modified search algorithms for the other databases at the end of this Appendix.

### Eligibility Criteria

#### Study Selection

We will include randomized controlled trials, case studies, observational studies, surveys, empirical evidence, systematic reviews, and meta-analysis.

All articles selected by our search algorithm will be imported to an Endnote library. Duplicates will be eliminated. The articles will first be evaluated based on abstracts, followed by a full-text article review. Both PIs will independently evaluate all abstracts and full-text articles.

#### Exclusion Criteria

We will exclude policy briefs, editorials, commentaries, reports, and books.

#### Time Period

The search window will be from January 1, 1970 to April 30, 2013. We will try to identify newer studies of high relevance that are published after the initial search is done by staying updated on the field and in contact with colleagues.

### Data Extraction

Data will be extracted on a standard, structured form (MS Excel) according to the PRISMA guidelines. A pilot will be developed to test the data extraction sheet. If insufficient data are captured or clarifications are necessary for the selected studies, we will contact the respective authors for further inquiries.

We will try to evaluate articles in line with the PICO (population, intervention, comparison, outcome) criteria as far as possible:

- Population: women throughout the life cycle, globally
- Interventions: all interventions that improve women’s health:

1. Health interventions
2. Non-health interventions:
   1. Social
   2. Political
   3. Environmental
   4. Economic

- Comparisons: Assess the rates of return to investments in women’s health in relation to standard investment in economic growth when possible, i.e., education, infrastructure
- Outcomes: Look at economic and health outcomes that are important indirect pathways for economic growth:

1. Economic outcomes
   1. Microeconomic (household/family) level: assets, income, purchasing power, savings, use of services, productivity, labor force participation, educational attainment, costs related to illness/health care services
   2. Macroeconomic (country) level: GDP, GNP, growth
2. Health
   1. Birth weight, mortality, morbidity, stunting, growth, cognitive development.

Articles with abstracts fulfilling these criteria will be reviewed in full text. Articles with too simplistic a methodology that do not report on methodology, or do not control for important covariables will be excluded, even if the outcome measures satisfy the inclusion criteria. The full selection process will be illustrated in a figure in final review.

### Role of Reviewers

Two independent reviewers (JHI and KHO) will first review the titles and abstracts of studies that meet the initial criteria for inclusion. The researchers will then review the full text of articles that cannot be evaluated based on title and abstract alone. If disagreement occurs concerning inclusion or exclusion of articles, we will first try to resolve this through in-depth discussion; if this fails, a third researcher (DB) will evaluate the paper, and the three researchers will make a final decision together.

### Data Synthesis

Findings will be focused on the identified pathways and summarized in text and tables for productivity, savings, education, fertility and intergenerational health. We will report total studies included. We will summarize the existing knowledge and current gaps in the literature.

### Assessment of Bias Risk

Reviewers will discuss potential bias concerns in the included articles during data synthesis and report potential bias in the final review.

# Final Search Strategy by Databases

#### PubMed/MEDLINE Database

("Economic Development"[Mesh] OR "Work"[Mesh:NoExp] OR "Employment"[Mesh:NoExp] OR "Salaries and Fringe Benefits"[Mesh:NoExp] OR "Income"[Mesh:NoExp] OR "Poverty/economics"[Mesh] OR "Poverty/prevention and control"[Mesh] OR earnings[tiab] OR economic benefit[tiab] OR economic consequences[tiab] OR economic contribution[tiab] OR economic development[tiab] OR economic growth[tiab] OR economic impact[tiab] OR economic improvement*[tiab] OR economic investment*[tiab] OR economic productivity[tiab] OR economic returns[tiab] OR economic situation[tiab] OR economic status[tiab] OR economic well being[tiab] OR formal labor[tiab] OR gross domestic product[tiab] OR gross national product[tiab] OR household consumption[tiab] OR income[tiab] OR incomes[tiab] OR informal labor[tiab] OR informal sector[tiab] OR labor force[tiab] OR labour force[tiab] OR labor market*[tiab] OR labour market*[tiab] OR labor participation[tiab] OR labour participation[tiab] OR macroeconomic*[tiab] OR macroeconomic[tiab] OR microcredit[tiab] OR micro credit[tiab] OR microeconomic*[tiab] OR micro economic[tiab] OR paid labor[tiab] OR paid labour[tiab] OR poverty alleviation[tiab] OR poverty reduction[tiab] OR reduce poverty[tiab] OR salary[tiab] OR salaries[tiab] OR savings[tiab] OR wages[tiab]) AND ("Mortality"[Mesh:NoExp] OR "Morbidity"[Mesh:noexp] OR "Child Mortality"[Mesh] OR "Infant Mortality"[Mesh] OR "Infanticide"[Mesh] OR "Malnutrition"[Mesh] OR "Maternal Mortality"[Mesh] OR "Perinatal Mortality"[Mesh] OR "Reproductive Health"[Mesh] OR "Women's Health"[Mesh] OR adolescent health[tiab] OR burden of disease[tiab] OR child health[tiab] OR children's health[tiab] OR child mortality[tiab] OR disease burden[tiab] OR female morbidity[tiab] OR female mortality[tiab] OR infant mortality[tiab] OR infanticide[tiab] OR malnutrition[tiab] OR maternal health[tiab] OR maternal morbidity[tiab] OR maternal mortality[tiab] OR mortality decline*[tiab] OR neonatal mortality[tiab] OR perinatal mortality[tiab] OR reproductive health[tiab] OR ((abortion[tiab] OR abortions[tiab]) AND (sex selective[tiab] OR sex biased[tiab] OR sex preference* [tiab] OR son preference[tiab] OR sex selection[tiab] OR sex selected[tiab] OR missing girls[tiab] OR gender imbalance[tiab] OR female feticide[tiab] OR female fetus*[tiab]) OR sexual health[tiab] OR undernutrition[tiab]) AND ("Female"[Mesh] OR "Women's Health"[Mesh] OR adolescent girls[tiab] OR adult women[tiab] OR elderly women[tiab] OR gender[tiab] OR older women[tiab] OR women's health[tiab] OR young women[tiab] OR maternal[tiab] OR female[tiab] OR female’s[tiab] OR females[tiab] OR women[tiab] OR women’s[tiab])

#### Excerpta Medica Database (Embase)

'economic development'/exp/mj OR 'poverty'/exp OR 'earnings' OR 'economic benefit' OR 'economic consequences' OR 'economic contribution' OR 'economic development'/exp OR 'economic growth'/exp OR 'economic impact' OR 'economic improvement' OR 'economic investment' OR 'economic investments' OR 'economic productivity' OR 'economic returns' OR 'economic situation' OR 'economic status' OR 'economic well being' OR 'formal labor' OR 'formal labour' OR 'gross domestic product'/exp OR 'gross national product'/exp OR 'household consumption' OR 'income'/exp OR 'incomes' OR 'informal labor' OR 'informal sector' OR 'labor force' OR 'labour force' OR 'labor market' OR 'labour market' OR 'labor participation' OR 'labour participation' OR 'macroeconomic' OR 'macro economic' OR 'microcredit' OR 'micro credit' OR 'microeconomic' OR 'micro economic' OR 'paid labor' OR 'paid labour' OR 'poverty alleviation' OR 'poverty reduction' OR 'reduce poverty' OR 'salary'/exp OR 'salaries' OR 'savings' OR 'wages' OR 'work'/exp AND ('mortality'/exp OR 'childhood mortality'/exp OR 'morbidity'/exp OR 'reproductive health'/exp OR 'women`s health'/exp OR 'sexual health'/exp OR 'adolescent health'/exp OR 'burden of disease' OR 'child health'/exp OR 'children`s health' OR 'child mortality'/exp OR 'disease burden' OR 'female morbidity' OR 'female mortality' OR 'infant mortality'/exp OR 'infanticide'/exp OR 'malnutrition'/exp OR 'maternal health'/exp OR 'maternal morbidity'/exp OR 'maternal mortality'/exp OR 'mortality decline' OR 'neonatal mortality'/exp OR 'perinatal mortality'/exp AND ('abortion'/exp OR 'abortions') AND ('sex selective' OR 'sex biased' OR 'sex preference' OR 'son preference' OR 'sex selection' OR 'sex selected' OR 'missing girls' OR 'gender imbalance' OR 'female feticide' OR 'female fetus') OR 'undernutrition'/exp) AND ('adolescent girls' OR 'adult women' OR 'elderly women' OR 'gender'/exp OR 'older women' OR 'women`s health'/exp OR 'young women' OR 'maternal' OR 'female'/exp OR 'female`s' OR 'females'/exp OR 'women'/exp OR 'women`s') AND [english]/lim AND [1970-2013]/py

#### Cochrane Database

'("Employment" OR "earnings" OR "economic benefit" OR "economic consequences" OR "economic contribution" OR "economic development" OR "economic growth" OR "economic impact" OR "economic improvement*" OR "economic investment*" OR "economic productivity" OR "economic returns" OR "economic situation" OR "economic status" OR "economic well being" OR "formal labor" OR "gross domestic product" OR "gross national product" OR "household consumption" OR "income" OR "incomes" OR "informal labor" OR "informal sector" OR "labor force" OR "labour force" OR "labor market*" OR "labour market*" OR "labor participation" OR "labour participation" OR "macroeconomic*" OR "macro economic" OR "microcredit" OR "micro credit" OR "microeconomic*" OR "micro economic" OR "paid labor" OR "paid labour" OR "poverty alleviation" OR "poverty reduction" OR "reduce poverty" OR "salary" OR "salaries" OR "savings" OR "wages") AND ("Mortality" OR "Morbidity" OR "Women's Health" OR "adolescent health" OR "burden of disease" OR "child health" OR "children's health" OR "child mortality" OR "disease burden" OR "female morbidity" OR "female mortality" OR "infant mortality" OR "infanticide" OR "malnutrition" OR "maternal health" OR "maternal morbidity" OR "maternal mortality" OR "mortality decline*" OR "neonatal mortality" OR "perinatal mortality" OR "reproductive health" OR (("abortion" OR "abortions") AND ("sex selective" OR "sex biased" OR "sex preference*" OR "son preference" OR "sex selection" OR "sex selected" OR "missing girls" OR "gender imbalance" OR "female feticide" OR "female fetus*")) OR "sexual health" OR "undernutrition") AND ("Female" OR "Women's Health" OR "adolescent girls" OR "adult women" OR "elderly women" OR "gender" OR "older women" OR "women's health" OR "young women" OR "maternal" OR "women" OR "female’s" OR "females" OR "women’s") in title abstract keywords from 1970 to 2013

#### EconLit Database

((contribution) OR AB,TI(economic development) OR AB,TI(economic growth) OR AB,TI(economic impact) OR AB,TI(economic improvement*) OR AB,TI (economic investment*) OR AB,TI(economic productivity) OR AB,TI(economic returns) OR AB,TI(economic situation) OR AB,TI(economic status) OR AB,TI(economic well being) OR AB,TI(employment) OR AB,TI(formal labor) OR AB,TI(gross domestic product) OR AB,TI(gross national product) OR AB,TI(household consumption) OR AB,TI(income) OR AB,TI(incomes) OR AB,TI( informal labor) OR AB,TI(informal sector) OR AB,TI(labor force) OR AB,TI(labour force) OR AB,TI(labor market*) OR AB,TI(labour market*) OR AB,TI(labor participation) OR AB,TI(labour participation) OR AB,TI(macroeconomic*) OR AB,TI(macro economic) OR AB,TI(microcredit) OR AB,TI(micro credit) OR AB,TI(microeconomic*) OR AB,TI(micro economic) OR AB,TI(paid labor) OR AB,TI(paid labour) OR AB,TI(poverty alleviation) OR AB,TI(poverty reduction) OR AB,TI(reduce poverty) OR AB,TI(salary) OR AB,TI(salaries) OR AB,TI(savings) OR AB,TI(wage*)) AND (AB,TI(mortality) OR AB,TI(morbidity) OR AB,TI(adolescent health) OR AB,TI(burden of disease) OR AB,TI(child health) OR AB,TI(children's health) OR AB,TI(child mortality) OR AB,TI(disease burden) OR AB,TI(female morbidity) OR AB,TI(female mortality) OR AB,TI(infant mortality) OR AB,TI(infanticide) OR AB,TI(malnutrition) OR AB,TI(maternal health) OR AB,TI(maternal morbidity) OR AB,TI(maternal mortality) OR AB,TI(mortality decline*) OR AB,TI(neonatal mortality) OR AB,TI(perinatal mortality) OR AB,TI(reproductive health) OR AB,TI(abortion*) OR AB,TI(sex-selective abortion*) OR AB,TI(sexual health) OR AB,TI(undernutrition)) AND (AB,TI(adolescent girls) OR AB,TI(adult women) OR AB,TI(elderly women) OR AB,TI(gender) OR AB,TI(older women) OR AB,TI(women's health) OR AB,TI(young women) OR AB,TI(female) OR AB,TI(female’s) OR AB,TI(females) OR AB,TI(women) OR AB,TI(women’s))

#### Web of Science Database

(("Employment" OR "earnings" OR "economic benefit" OR "economic consequences" OR "economic contribution” OR “economic development” OR “economic growth” OR “economic impact” OR “economic improvement?” OR “economic investment?” OR “economic productivity” OR “economic returns” OR “economic situation” OR “economic status” OR “economic well being” OR “formal labor” OR “gross domestic product” OR “gross national product” OR “household consumption” OR “income” OR “incomes” OR “informal labor” OR “informal sector” OR “labor force” OR “labour force” OR “labor market*” OR “labour market?” OR “labor participation” OR “labour participation” OR “macroeconomic?” OR “macro economic” OR “microcredit” OR “micro credit” OR “microeconomic*” OR “micro economic” OR “paid labor” OR “paid labour” OR “poverty alleviation” OR “poverty reduction” OR “reduce poverty” OR “salary” OR “salaries” OR “savings” OR “wages”)

AND ("Mortality" OR "Morbidity" OR "Women's Health" OR “adolescent health” OR “burden of disease” OR “child health” OR “children's health” OR “child mortality” OR “disease burden” OR “female morbidity” OR “female mortality” OR “infant mortality” OR “infanticide” OR “malnutrition” OR “maternal health” OR “maternal morbidity” OR “maternal mortality” OR “mortality decline?” OR “neonatal mortality” OR “perinatal mortality” OR “reproductive health” OR ((“abortion” OR “abortions”) AND (“sex selective” OR “sex biased” OR “sex preference*” OR “son preference” OR “sex selection” OR “sex selected” OR “missing girls” OR “gender imbalance” OR “female feticide” OR “female fetus*”)) OR “sexual health” OR “undernutrition”)

AND ("Female" OR "Women's Health" OR “adolescent girls” OR “adult women” OR “elderly women” OR “gender” OR “older women” OR “women's health” OR “young women” OR “maternal” OR “women” OR “female’s” OR “females” OR “women’s”))

#### Cumulative Index to Nursing and Allied Health Literature (CINAHL) Database

AB ((“earning” OR “economic benefit*” OR “economic consequences” OR “economic contribution” OR “economic development” OR “economic growth” OR “economic impact” OR “economic improvement*” OR “economic investment*” OR “economic productivity” OR “economic returns” OR “economic situation” OR “economic status” OR “economic well being” OR “formal labor” OR “gross domestic product” OR “gross national product” OR “household consumption” OR “income*” OR “informal labor” OR “informal sector” OR “labor force” OR “labour force” OR “labor market*” OR “labour market*” OR “labor participation” OR “labour participation” OR “macroeconomic*” OR “macro economic” OR “microcredit” OR “micro credit” OR “microeconomic*” OR “micro economic” OR “paid labor” OR “paid labour” OR “poverty alleviation” OR “poverty reduction” OR “reduce poverty” OR “salary” OR “salaries” OR “savings” OR “wage*” OR “employment”)) AND AB ((“mortality” OR “morbidity” OR “adolescent health” OR “burden of disease” OR “child health” OR “children's health” OR “child mortality” OR “disease burden” OR “female morbidity” OR “female mortality” OR “infant mortality” OR “infanticide” OR “malnutrition” OR “maternal health” OR “maternal morbidity” OR “maternal mortality” OR “mortality decline*” OR “neonatal mortality” OR “perinatal mortality” OR “reproductive health” OR “sex-selective abortion*” OR “abortion*” OR “sexual health” OR “undernutrition”)) AND AB ((“adolescent girl*” OR “adult women” OR “elderly women” OR “gender” OR “older women” OR “women's health” OR “young women” OR “female” OR “female’s” OR “females” OR “women” OR “women’s”))

#### National Bureau of Economic Research (NBER) Working Paper Database

Manual search (due to no download reference opportunities and difficult to perform systematically)

Search strategy: We left out economic search terms, as it is an economic database and the search strategy was focused on health terms.

NBER Full Author/Title Search

neonatal health

neonatal mortality

infant health

infant mortality

child health

child mortality

adolescent health

maternal health

maternal mortality

female mortality

adult mortality

burden of disease

disease burden

fertility

women’s health

morbidity

abortion

mortality decline

malnutrition, undernutrition, nutrition

female

#### Latin American and Caribbean Health Sciences Literature (LILACS) Database

(AB:"economics" OR AB:"economic development" OR AB: "macroeconomics" OR AB:"microeconomics" OR AB:"household consumption" OR AB:"home economics" OR AB:"economic conditions" OR AB:"poverty" OR AB:"employment" OR AB:"income" OR AB:"gross national product" OR AB:"labor force" OR AB:"labour force") AND (AB:"Mortality" OR AB:"Morbidity" OR AB:"Child Mortality" OR AB:"Infant Mortality" OR AB:"Infanticide" OR AB:"Malnutrition" OR AB:"Maternal Mortality" OR AB:"Perinatal Mortality" OR AB:"Reproductive Health" OR AB:"Women's Health" OR AB:"adolescent health" OR AB:"burden of disease" OR AB:"child health" OR AB:"children's health" OR AB:"child mortality" OR AB:"disease burden" OR AB:"female morbidity" OR AB:"female mortality" OR AB:"infant mortality" OR AB:"infanticide" OR AB:"malnutrition" OR AB:"maternal health" OR AB:"maternal morbidity" OR AB:"maternal mortality" OR AB:"mortality decline" OR AB:"neonatal mortality" OR AB:"perinatal mortality" OR AB:"reproductive health" OR ((AB:"abortion" OR AB:"abortions") AND (AB:"sex selective" OR AB:"sex biased" OR AB:"sex preference" OR AB:"son preference" OR AB:"sex selection" OR AB:"sex selected" OR AB:"missing girls" OR AB:"gender imbalance" OR AB:"female feticide" OR AB:"female fetus")) OR AB:"sexual health" OR AB:"undernutrition") AND (AB:"adolescent girls" OR AB:"adult women" OR AB:"elderly women" OR AB:"gender" OR AB:"older women" OR AB:"women's health" OR AB:"young women" OR AB:"maternal" OR AB:"female" OR AB:"female’s" OR AB:"females" OR AB:"women" OR AB:"women’s")

#### POPLINE Database

Key word search:

Keyword I

(Economics,Economic Development,Macroeconomic Factors,Microeconomic Factors,Home Economics,Economic Conditions,Economic Factors,Poverty,Employment,Income,Gross National Product,Labor Force)

Keyword II

(Women's Health,Maternal Health,Child Health,Malnutrition,Abortion,Infanticide,Child Mortality,Adolescent Health,Perinatal Mortality,Infant Health,Infant Mortality,Maternal Mortality,Mortality,Morbidity,Neonatal Mortality,Reproductive Health,Mortality Changes)

Keyword III

(Child Female,Adolescents Female,Adults,Older Adults,Older Adults 80 and Over,Women,Women's Health,Gender Issues)

#### Genderwatch Database

(TI,AB(earnings) OR TI,AB(economic benefit) OR TI,AB(economic consequences) OR TI,AB(economic contribution) OR TI,AB(economic development) OR TI,AB(economic growth) OR TI, AB(economic impact) OR TI,AB(economic improvement) OR TI,AB(economic productivity) OR TI,AB(economic returns) OR TI,AB(economic situation) OR TI,AB(economic status) OR TI,AB(economic well being) OR TI,AB(economic investment*) OR TI,AB(employment) OR TI,AB(formal labor) OR TI,AB(gross domestic product) OR TI,AB(gross national product) OR TI,AB(household consumption) OR TI,AB(income) OR TI,AB(incomes) OR TI,AB(informal labor) OR TI,AB(informal sector) OR TI,AB(labor force) OR TI,AB(labour force) OR TI,AB(labor market) OR TI,AB(labour market) OR TI,AB(labor participation) OR TI,AB(labour participation) OR TI,AB(macroeconomic) OR TI,AB(macro economic) OR TI, AB(microcredit) OR TI,AB(micro credit) OR TI,AB(microeconomic) OR TI, AB(micro economic) OR TI,AB(paid labor) OR TI,AB(paid labour) OR TI,AB(poverty alleviation) OR TI,AB( poverty reduction) OR TI,AB(reduce poverty) OR TI,AB(salary) OR TI,AB(salaries) OR TI,AB(savings) OR TI,AB(wage*)) AND (TI,AB(mortality) OR TI,AB(Morbidity) OR TI,AB(Women's Health) OR TI,AB(adolescent health) OR TI,AB(burden of disease) OR TI,AB(child health) OR TI,AB(children's health) OR TI, AB(child mortality) OR TI,AB(disease burden) OR TI,AB(female morbidity) OR TI,AB(female mortality) OR TI,AB(infant mortality) OR TI,AB(infanticide) OR TI,AB(malnutrition) OR TI,AB(maternal health) OR TI,AB(maternal morbidity) OR TI,AB(maternal mortality) OR TI, AB(mortality decline*) OR TI,AB(neonatal mortality) OR TI,AB(perinatal mortality) OR TI,AB(reproductive health) OR TI,AB(sex-selective abortion*) OR TI,AB(abortion*) OR TI,AB(sexual health) OR TI,AB(undernutrition)) AND (TI,AB(Female) OR TI,AB(Women's Health) OR TI,AB(adolescent girls) OR TI,AB(adult women) OR TI,AB(elderly women) OR TI,AB(gender) OR TI,AB(older women) OR TI,AB(women's health”) OR TI, AB(young women) OR TI,AB(maternal) OR TI,AB(women) OR TI,AB(female’s) OR TI,AB(females) OR TI,AB(women’s))
